# Intelligent Fish-Inspired Foraging of Swarm Robots with Sub-Group Behaviors Based on Neurodynamic Models

**DOI:** 10.3390/biomimetics9010016

**Published:** 2024-01-01

**Authors:** Junfei Li, Simon X. Yang

**Affiliations:** School of Engineering, University of Guelph, 50 Stone Road East, Guelph, ON N1G2W1, Canada; jli64@uoguelph.ca

**Keywords:** swarm robots, bio-inspired algorithms, foraging behaviors, fish-inspired algorithm, neurodynamic models

## Abstract

This paper proposes a novel intelligent approach to swarm robotics, drawing inspiration from the collective foraging behavior exhibited by fish schools. A bio-inspired neural network (BINN) and a self-organizing map (SOM) algorithm are used to enable the swarm to emulate fish-like behaviors such as collision-free navigation and dynamic sub-group formation. The swarm robots are designed to adaptively reconfigure their movements in response to environmental changes, mimicking the flexibility and robustness of fish foraging patterns. The simulation results show that the proposed approach demonstrates improved cooperation, efficiency, and adaptability in various scenarios. The proposed approach shows significant strides in the field of swarm robotics by successfully implementing fish-inspired foraging strategies. The integration of neurodynamic models with swarm intelligence not only enhances the autonomous capabilities of individual robots, but also improves the collective efficiency of the swarm robots.

## 1. Introduction

**Figure 1:**
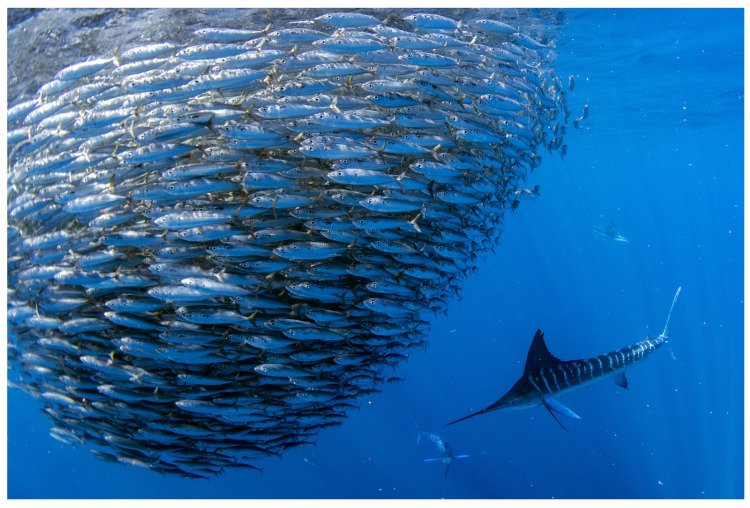
The group behavior of fish schools (credit: iStock).

The collective behavior exhibited by fish schools is a remarkable example of biological organization in natural environments [1]. Fish schools are usually made up of hundreds or thousands of individual fish, which show a high degree of coordination in their movements [2]. Collective behavior helps to make foraging efficient, offers protection against predators, and helps to navigate complex environments, as shown in Figure 1. Furthermore, dynamic changes in the shape and direction of schools are implemented by simple local interactions between individual fish, which has inspired many researchers to develop algorithms and systems that govern similar efficient collective movement [3].

Recently, there has been a trend to develop new systems, algorithms, and robotic strategies inspired by the behavior and intelligence of fish [4,5]. Chen et al. [6] proposed an innovative design in the realm of robotic fish, which incorporated a high-frequency oscillation mechanism paired with a compliant and passive system. The proposed robotic system showed rapid swimming capabilities and closely mimicked the learning patterns of the fish. Weber et al. [7] introduced the optimal placement of the sensor in artificial swimmers, that demonstrated that the follower distribution is similar to the neuromast distribution in fish. Hannard et al.  [8] introduced novel robotic materials based on inspiration from mechanical modeling and testing of fish fins, which are capable of achieving significant morphing amplitudes and robust grasping forces. By mimicking intelligent schools of fish, swarm robots can perform complex tasks collectively without central control. The designed swarm robots collaborate with each other based on local interactions, responding instantly to their neighbors [9]. Cioarga et al. [10] introduced collision-free fountain maneuvers and flash expansion variations for mobile robots. Similarly, Berlinger et al. [11] adapted the fountain maneuver model to an underwater robotic platform, ensuring a consistently visible angle for predators. Novák et al. [12] presented an animal-inspired and rapid escape technique that allows swarm robots to avoid dynamic obstacles. Min and Wang [13] introduced a fish-inspired escape algorithm that enables rapid predator evasion and obstacle avoidance. Li [14] proposed a self-adaptive collective escape approach inspired by the group escape behavior of fish. The potential applications of these fish-inspired robotic systems are vast [15]. For instance, fish-inspired systems can be used in deep-sea exploration in extreme pressure environments [16]. In addition, fish-inspired systems can finish emergency search and rescue missions to guarantee the safety of human life [17]. Moreover, fish-inspired systems can also achieve cooperative transportation in underwater environments [18].

Foraging behaviors are highly efficient strategies that exploit food resources through collective search patterns and local interactions. However, translating efficient foraging behaviors into robotic systems is a significant challenge. Song et al. [19] proposed a foraging approach of swarm robots based on virtual pheromones through the dynamics of neural networks. Pang et al. [20] proposed a foraging approach based on the dynamic calculation of the food stimulus and the number of resting robots, and on obstacle avoidance in the last foraging task. Lee et al. [21] proposed a self-organizing allocation method of swarm robots for sequential foraging tasks based on the response threshold model. Research inspired by the foraging behavior of fish schools is notably scarce [22]. Li et al. [23] proposed an artificial fish swarm algorithm (AFSA) to use the local searches of individual fish for finding the global optimum solution. Foraging behavior is one of the simulated behaviors in the AFSA. The AFSA and many hybrid models have been studied to solve real-world problems, such as data clustering, image segmentation, and parameter optimization [24]. However, most AFSA-based approaches are used to solve optimization problems. Foraging behavior is merely one optimization strategy, instead of the collective control of robots. Connor et al. [25] proposed a fish-inspired robotic algorithm (FIRA) to mimic foraging behavior, inspired by schooling fish. However, their method does not consider the scenario where fish schools can be divided into different sub-groups, leading to lower efficiency in multi-target problems. Berlinger et al. [26] developed a potential-field-based model to emulate collective behavior in underwater robots within three-dimensional spaces. However, they only considered collision-free scenarios, which might not accomplish complex tasks in the real-world environment. Compared to traditional foraging methods, the collective behavior of fish schools is characterized by the following:

(1) The foraging behavior of fish schools is implemented based on interactions between individuals, rather than global pheromones or propagation [1,11,26];
(2) During the foraging process, fish schools maintain appropriate distances between individuals and shape formation [13,26];
(3) Fish schools exhibit adaptive behavior to accommodate changes in complex environments [1,2].

The purpose of this paper is to enable swarm robots with several characteristics of fish foraging behavior. In traditional foraging approaches, the foraging behavior of fish schools is implemented based on global communications. In addition, robotic systems typically lack the ability to adapt to environmental changes. Furthermore, robotic systems do not consider the sub-group scenario, where fish schools can be divided into different small groups during the foraging process. In the proposed approach, a bio-inspired neural network (BINN) is proposed to generate virtual forces, and a neurodynamic model is incorporated to enhance the self-adaptive ability of swarm robots. In addition, a self-organizing map (SOM) is proposed to facilitate sub-group behavior within the swarm robots, which enables the robotic swarm to mimic the natural division and regrouping of fish schools during foraging. The simulation results show the efficacy of the proposed approach in ensuring safe, efficient, and self-adaptive cooperation among autonomous robots in many environments.

The main contributions of this paper are summarized as follows:

(1) A novel collective foraging approach is proposed for swarm robots in changing environments. The proposed approach takes inspiration from the locally interactive group behavior of fish.
(2) A novel approach to collision-free virtual forces is proposed to guide swarm robots based on a neurodynamics model. During the foraging process, the swarm robots have self-adaptive ability to accommodate changes in complex environments.
(3) An SOM algorithm is proposed to enable the sub-group behavior of the swarm robots, which enables the swarm robots to dynamically adjust their shape in complex environments.

This paper is organized as follows. Section 2 gives the description of the problem. Section 3 describes the proposed approaches. Section 4 shows the simulation results. Section 5 discusses the characteristics of the neurodynamic model. In Section 6, the results are briefly summarized.

## 2. Problem Description

For a swarm of *m* robots, their location in the 2D Cartesian workspace *W*, can be uniquely determined by the spatial position $p_{i}=\left(x_{i},y_{i}\right)$, i=1,…,m. The maximum speed the robot can achieve during the foraging process is denoted by $V_{max}>0$. It is assumed that each robot functions as an omnidirectional entity and is capable of altering its movement direction instantaneously without delay. The next location of the *i*-th robot at time instant t+1 can be given as

(1) $${\left(x_{i}\right)}_{t+1}={\left(x_{i}\right)}_{t}+v_{i}\Delta t\cos{\left(\theta_{i}\right)}_{t}$$

(2) $${\left(y_{i}\right)}_{t+1}={\left(y_{i}\right)}_{t}+v_{i}\Delta t\sin{\left(\theta_{i}\right)}_{t}$$

where $v_{i}\leq V_{max}$ is the current speed of the robot; $\theta_{i}$ is the moving direction of the robot; and Δt is the unit time interval. In addition, there is a sequence of obstacles in *W*. Let O be an obstacle scenario. The collision-free area pertaining to O can be defined as $O_{\mathrm{free}\;}:=\left\{\left(x,y\right)\in R^{2}:\Gamma >1\right\}$, where

(3) $$\Gamma =\frac{{\left(x-x_{o}\right)}^{2}+{\left(y-y_{o}\right)}^{2}}{\lambda_{o}}$$

where $\left(x_{o},y_{o}\right)$ is the center of the obstacle, and $\lambda_{o}$ is the size of the obstacle. The regions meeting Γ=1, Γ>1, or Γ<1 denote the surface, exterior, and interior of the obstacle, respectively. The position of the target can be denoted by $T_{h}=\left(x_{h},y_{h}\right)$, h=1,…,q. The position of target $T_{h}$ is known to the robots. The target is foraged by the robot if the distance between the target and the robot is less than the capture distance $d_{f}>0$.

On the basis of the characteristics of fish schools, the foraging of a swarm of robots should be able to meet the following requirements.

(1) The foraging behavior is based on local interactions between individual robots.
(2) The swarm robots should be self-adaptive during the foraging process.
(3) The robots should maintain the desired distance $R_{d}$ from their neighbors.

Therefore, the fish-inspired foraging studied in this paper can be described as: for a group of *m* robots and given the initial positions of the robots $p_{i}\left(0\right)$ with i=1,…,m, since the *h*-th target exists in *W*, the swarm robot begins to forage and generate collision-free trajectories, that is, $P\in O_{\mathrm{free}\;}$, until any robots achieve a foraging distance $d_{f}$ to all targets in $T_{h}$. During the foraging process, the swarm robots are required to maintain the desired distance $R_{d}$ from the neighboring robots and to be self-adaptive to adapt to changing environments.

## 3. Proposed Approaches

In this section, a fish-inspired system is introduced for the foraging behavior of robots and a sub-groups mechanism based on the SOM algorithm is proposed. In addition, a neural network structure is proposed to generate collision-free virtual forces and improve the self-adaptive ability to swam robots.

### 3.1. Fish-Inspired Foraging Behavior

Fish schools exhibit highly efficient group behavior through simple individual interactions. In this paper, the foraging behavior is modeled as a mode-transition process. Each robot has two modes: *foraging* mode and *pilot* mode. The behavior of the robot is determined by the current mode. Once targets exist in the environment, the foraging process begins. The foraging process can be summarized as follows:

(1) Based on the number of targets, swarm robots are divided into different subdivision groups using the SOM algorithm. Within each subdivision group, one robot is selected for *pilot* mode, while the other robots switch to *foraging* mode;
(2) The *pilot* robot requests the coordinates and status of all the *foraging* robots in the subdivision group;
(3) After the group of swarm robots is constructed, all the robots in the same subdivision group begin to move cooperatively to the target.

Note that the fish organization was considered an egalitarian organization in the past several decades. Therefore, traditional fish-inspired approaches assumed that swarm robots are an egalitarian organization [11,13,26]. However, current studies have found that hierarchical organization might exist in some species of fish [1]. The purpose of this paper is to use new ideas to design fish-inspired swarm robots. In addition, every robot can be designated as a temporary *pilot* robot, which means the system operates in a decentralized manner.

### 3.2. Sub-Group Mechanism Based on SOM

**Figure 2:**
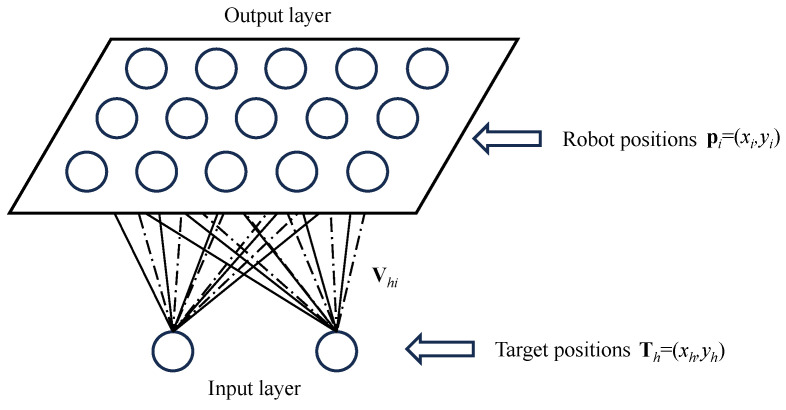
Structure of SOM neural network.

When a target is placed within the environment, the foraging process begins. In scenarios where a single target is present, the robots bypass the implementation of group division. In scenarios with multiple targets, it requires the strategic organization of swarm robots into distinct groups. As shown in Figure 2, the SOM neural network is made up of two layers. The input layer consists of two neurons, which represent the coordinates of the targets $T_{h}=\left(x_{h},y_{h}\right)$. The output layer is assigned to map the locations of the robots $p_{i}=\left(x_{i},y_{i}\right)$. The connection weights between the neurons of the input layer and the output layer are determined by a weight vector $V_{hi}$, which is initially calibrated according to the coordinates of the robots.

The SOM decomposes the entire issue into sub-problems. In each iteration, with a specified target input, the process encompasses three stages. Initially, the winner is identified. The second is the determination of its neighboring neurons. The third stage involves altering the weights of both the winner and its neighbors. This sequence is reiterated until a stabilization of all weights is observed. Therefore, swarm robots gradually form distinct groups and align themselves based on the evolving weight changes relative to the location of targets. For a given robot as an input, the output neurons compete to be the winner according to a specified criterion, described as [27]

(4) $$\begin{array}{c} N_{i}⇐\min\left\{D_{hi},h=1,\ldots ,q;i=1,\ldots ,m\right.\} \end{array}$$

where $D_{hi}=\left|T_{h}-p_{i}\right|$ is the weighted distance and $N_{i}$ denotes that the *i*-th neuron is the winner neuron from the *h* input neurons. Once the winner is identified, the subsequent phase involves designing the neighborhood function. The neighborhood function denotes the impact of the robot location on the winner and adjacent neurons, which dictates the attraction strength. The influence exerted by the winner is strongest and progressively diminishes with the decreasing proximity of other neurons, whereas it does not affect those outside the designated neighborhood. The neighborhood function is characterized as

(5) $$\begin{array}{c} f\left(d_{j},G\right)=\left\{\begin{array}{cc} e^{-d_{j}^{2}/G^{2}\left(t\right)}, & \;\mathrm{if}\;d_{j}<r\\ 0, & \;\mathrm{otherwise}\; \end{array}\right. \end{array}$$

where $d_{j}=||j-N_{i}\left|\right|,j=1,2,...,J$ represents the distance between the *j*-th neuron and the winner neuron $N_{i}$ and *r* defines the neighborhood range. Function $G\left(t\right)={\left(1-\alpha \right)}^{t}G_{0}$ is a nonlinear function, where *t* denotes the iteration count, and α is the change rate that influences the computation time.

After the selection of the winner neuron and its neighbors, the ensuing step involves adjusting the weights of the winner neuron with neighboring neurons. The update rule is described as

(6) $$p_{i}\left(t+1\right)=\left\{\begin{array}{cc} T_{h}\left(t\right), & \;\mathrm{if}\;D_{hi}\leq d_{f}\\ p_{i}\left(t\right)+\gamma f\left(d_{j},G\right)\times \left(T_{h}\left(t\right)-p_{i}\left(t\right)\right), & \;\mathrm{otherwise}\; \end{array}\right.$$

where γ is the learning rate and η is a small constant. The introduction of $d_{f}$ can significantly reduce the computational duration of the algorithm [27]. The adjustment of weights is influenced not solely by the initial distance between the winner, its neighbors, and the input target neuron, but also by the neighborhood function and the learning rate. Upon stabilization of the weight vectors $V_{hi}$, the swarm of robots is then divided into distinct groups based on the target. Within each group, one robot is randomly selected to serve as the *pilot* mode, while the remaining robots change to *foraging* mode.

### 3.3. Virtual Force Generation Based on BINN

**Figure 3:**
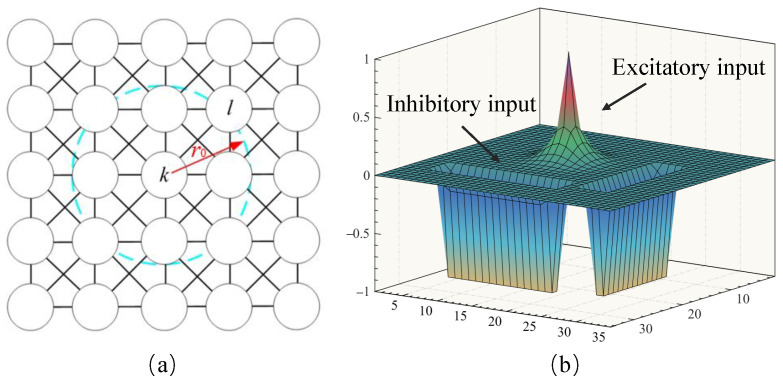
Examples of the bio-inspired neural network: (**a**) Structure of the neural network with only local connections. (**b**) The dynamic landscape of neural activity for a 35×35 neural network.

After the sub-groups of swarm robots are constructed, all the robots in the sub-group begin to forage the same target. The foraging behavior can be considered a unique form of robot path planning that necessitates the cooperative movement of all robots in a group towards a common target. In this paper, a neural network architecture is proposed for representing the environment. Environmental positions are mapped one-to-one to corresponding neuronal positions, as shown in Figure 3a. Note that each neuron has only local lateral connection to its neighboring neurons and responds only to the stimulus within its receptive field with a radius of $r_{0}$. In the proposed BINN, the dynamics of neural activity in the neural network is characterized by a shunting equation.

The shunting equation was developed by Grossberg [28] based on Hodgkin and Huxley’s model [29]. The shunting equation can be written as

(7) $$\frac{d\zeta_{k}}{dt}=-A\zeta_{k}+\left(B-\zeta_{k}\right)S_{k}^{e}-\left(D+\zeta_{k}\right)S_{k}^{i}$$

where $\zeta_{k}$ represents the neural activity of the *k*-th neuron; $S_{k}^{e}$ and $S_{k}^{i}$ denote the excitatory and inhibitory inputs, respectively; *A* refers to the passive decay rate; *B* and *D* are the upper and lower bounds of neural activity, respectively. This concept has been foundational in the development of various robotic navigation and control algorithms based on the shunting model [30,31]. The neural activity for the *k*-th neuron is written as

(8) $$\begin{array}{cc} \frac{d\zeta_{k}}{dt}=-A\zeta_{k} & +\left(B-\zeta_{k}\right)\left({\left[I_{k}\right]}^{+}+\sum_{l=1}^{n}w_{kl}{\left[\zeta_{l}\right]}^{+}\right)\\ \; & -\left(D+\zeta_{k}\right))\left({\left[I_{k}\right]}^{-}+\sum_{l=1}^{n}v_{kl}{\left[\zeta_{l}-\sigma \right]}^{-}\right) \end{array}$$

where $\zeta_{l}$ represents the neural activity of neighboring neurons to the *k*-th neuron; *n* represents the amount of neighboring neurons to the *k*-th neuron; ${\left[a\right]}^{+}$ is defined as ${\left[a\right]}^{+}=\max\left\{a,0\right\}$; ${\left[a\right]}^{-}$ is defined as ${\left[a\right]}^{-}=\max\left\{-a,0\right\}$; and σ is the threshold of the inhibitory lateral neural connections. The connection weights, $w_{kl}$ and $v_{kl}$, are defined as

(9) $$w_{kl}=f\left(|kl|\right)=\left\{\begin{array}{cc} \mu /|kl|, & 0<|kl|\leq r_{0}\\ 0, & |kl|>r_{0} \end{array}\right.$$

and

(10) $$v_{kl}=\beta w_{kl},$$

respectively, where β is a positive constant, β∈[0,1]; |kl| represents the Euclidean distance between the *k*-th neuron and the *l*-th neuron; and μ is a positive constant. The excitatory signal $S_{k}^{e}$ promotes positive neural activity, with the term $\sum_{l=1}^{n}w_{kl}{\left[\zeta_{l}\right]}^{+}$ facilitating the propagation of neural activity throughout the network. Conversely, the inhibitory signal $S_{k}^{i}$ induces negative neural activity, and the term $\sum_{l=1}^{n}v_{kl}{\left[\zeta_{l}-\sigma \right]}^{-}$ confines neural activity to a localized region due to the threshold σ. As a result, while $S_{k}^{e}$ exerts a global influence on the entire neural network, $S_{k}^{i}$ impacts only a limited area, as shown in Figure 3b. As shown in Algorithm 1, the key factors here are the size of the environment and the computations performed for each neuron. The overall computational complexity of the algorithm can be estimated as $O(N_{x}\times N_{y}\times {\left(2r_{0}+1\right)}^{2})$. This means the complexity of the algorithm is linearly related to the size of the environment grid and quadratically related to the choice of the radius $r_{0}$ defining the neighborhood. If $r_{0}$ is a small constant, then the complexity can be approximated as $O(N_{x}\times N_{y})$, linear to the size of the environment grid. In this study, the value of $r_{0}$ is a constant value ($r_{0}=\sqrt{2}$). Therefore, the computational complexity is $O(N_{x}\times N_{y})$, which can be approximated as $O(N^{2})$.
**Algorithm 1:** Computing Dynamic Landscape of Neural Activity
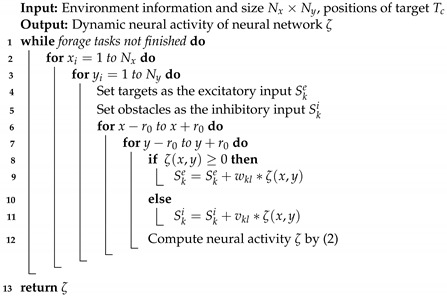


In this paper, attractive and repulsive forces are generated though the dynamics of neural activity. The external input $I_{k}$ fluctuates in accordance with the generation of force. When generating attractive force, the external input is $I_{att}$. Based on the modeling of the fish-inspired behavior, *foraging* robots need to track the *pilot* robot. Thus, the external input $I_{att}$ is defined as

(11) $$I_{att}=\left\{\begin{array}{cc} E, & \mathrm{if}\;\mathrm{it}\;\mathrm{is}\;a\;\mathrm{Pilot}\;\mathrm{robot}\\ -E, & \mathrm{if}\;\mathrm{it}\;\mathrm{is}\;\mathrm{an}\;\mathrm{obstacle}\\ 0, & \mathrm{otherwise} \end{array}\right.$$

where *E* is a positive constant. When the corresponding position aligns with a *pilot* robot, its external input assumes a significantly positive value. Conversely, if the position correlates with an obstacle, the external input takes on a substantially negative value. The command neuron for the attractive force can be defined as

(12) $$\begin{array}{ccc} P_{att}⇐\zeta_{P_{att}}=\max & \left\{\zeta_{l},l=1,2,...,n\right\} & \end{array}$$

where $P_{att}$ denotes the command neuron of the attractive force within the neural network; $x_{P_{att}}$ represents the neural activity of this command neuron. According to (12), the robot continuously searches for the maximum neural activity among its neighboring positions. As the robot moves to a new location, this location becomes its current position. The attractive force, denoted as $\bar{f_{A}\left(k,l\right)}$, is defined as

(13) $$\bar{f_{A}\left(k,l\right)}=C_{A}\frac{P_{att}-P_{c}}{∥P_{att}-P_{c}∥}$$

where $C_{A}$ represents a positive constant; and $P_{c}$ indicates the robot’s current position. Given the neuron representing an obstacle possesses a negative activity value, the robot can avoid selecting the obstacle neuron as its next position. When generating a repulsive force, the external input is $I_{rep}$. Based on the modeling of fish-inspired behavior, swarm robots are required to maintain the desired distance $R_{d}$ from each other. Thus, the external input $I_{rep}$ is defined as

(14) $$I_{rep}=\left\{\begin{array}{cc} E, & \mathrm{if}\;\mathrm{it}\;\mathrm{is}\;a\;\mathrm{neighbor}\;\mathrm{robot}\\ -E, & \mathrm{if}\;\mathrm{it}\;\mathrm{is}\;\mathrm{an}\;\mathrm{obstacle}\\ 0, & \mathrm{otherwise}. \end{array}\right.$$

When a corresponding position matches a neighboring robot, its external input is assigned a significantly positive value. Conversely, if the corresponding position relates to an obstacle, the external input is set to a notably negative value. The command neuron responsible for the repulsive force is defined as

(15) $$\begin{array}{ccc} P_{rep}⇐\zeta_{P_{rep}}=\min & \left\{\zeta_{l},l=1,2,...,n;\zeta_{l}\geq 0\right\} & \end{array}$$

where $P_{rep}$ represents the command neuron of the robot; and $x_{P_{rep}}$ represents the neural activity of the command neuron of the repulsive force. The repulsive force of the *foraging* robot $\bar{f_{R}\left(k,l\right)}$ can be defined as

(16) $$\bar{f_{R}\left(k,l\right)}==\left\{\begin{array}{cc} C_{R}\frac{P_{rep}-P_{c}}{∥P_{rep}-P_{c}∥} & \;\mathrm{if}\;0<D\left(k,l\right)\leq R_{d}\\ \bar{0}, & \;\mathrm{if}\;D\left(k,l\right)>R_{d} \end{array}\right.$$

where $C_{R}$ is a positive constant; D(k,l) is the distance between two neighboring robots *k* and *l*. The repulsive force exerted by a *foraging* robot becomes active only when the distance to neighboring robots is less than $R_{d}$. Consequently, the robot persistently seeks the lowest yet positive neural activity within its vicinity.

In the foraging process, swarm robots must exhibit self-adaptivity to their environments, entailing the dynamic adjustment of movement parameters in response to environmental conditions. The resultant force exerted by each robot is formulated as

(17) $$\bar{F_{RS}}=\alpha_{A}\sum_{l\in N\left(I\right),h_{l}<h_{k}}\bar{f_{A}\left(k,l\right)}+\alpha_{R}\sum_{l\in N(I)}\bar{f_{R}\left(k,l\right)}$$

where $\alpha_{A}$ and $\alpha_{R}$, $0\leq \alpha_{A},\alpha_{R}\leq 1$, and $\alpha_{A}+\alpha_{R}=1$, are the self-adaptive weights of the attractive and repulsive forces, respectively. The self-adaptive motion is to dynamically adjust the proper ratio of $\alpha_{A}$/$\alpha_{R}$ to adapt to the environmental changes. As shown in Algorithm 2, the dynamic neural activity is incorporated into the adjustment of the $\alpha_{A}$/$\alpha_{R}$ ratio. The stride lengths of adjustment Δ can be defined as

(18) $$\Delta =\left\{\begin{array}{cc} +U, & \mathrm{if}\;\mathrm{Avr}\left(i\right)+\sum_{l=1}^{n}{\left[\zeta_{l}\right]}^{-}>R_{d}\\ -U, & \mathrm{if}\;\mathrm{Avr}\left(i\right)+\sum_{l=1}^{n}{\left[\zeta_{l}\right]}^{-}\leq R_{d} \end{array}\right.$$

where *U* is a small constant. The function Avr(i) denotes the average neighboring distance of robot *i*. Function ${\left[a\right]}^{-}=\max\left\{-a,0\right\}$ denotes the sum of neighboring negative neural activity. If no obstacles are in proximity to the robots, the term representing the neural activity effect, $\sum_{l=1}^{n}{\left[\zeta_{l}\right]}^{-}$, should equate to 0. Therefore, robots adjust the ratio depending on whether their average neighboring distance is less than or exceeds $R_{d}$. If there are obstacles in proximity to the robots, $\sum_{l=1}^{n}{\left[\zeta_{l}\right]}^{-}$ is a large positive value. Thus, the attraction effect continuously increases, which ensures that robots remain connected with each other to bypass obstacles.
**Algorithm 2:** Neurodynamics-based self-adaptive mechanism
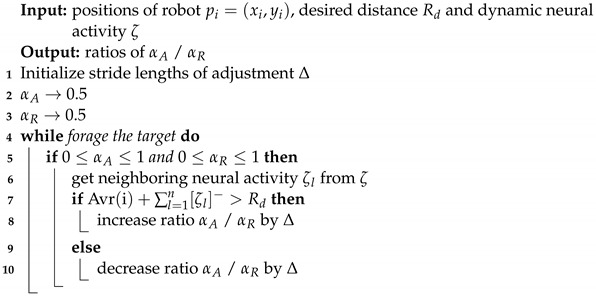


The virtual forces derived from the BINN are utilized to identify collision-free positions for subsequent movements. Nonetheless, the timing of these movements is contingent upon the robot’s velocity. The magnitude of $\bar{F_{RS}}$ determines the velocity $v_{k}$ of the robot. While the potential magnitude of $\bar{F_{RS}}$ ranges from 0 to +∞, the velocity $v_{k}$ is confined within a specific range, from 0 to a maximum velocity $V_{max}$. Consequently, there is a need to map the magnitude of $\bar{F_{RS}}$ onto a finite velocity scale. The velocity of the robot $v_{k}$ is given by

(19) $$v_{i}=\arctan\left(\left|\bar{F_{RS}}\right|\right)\times \left(2/\pi \right)\times V_{\max}$$

where arctan() is the trigonometric function. The above nonlinear mapping has been used for the collective motion [32]. The velocity $v_{i}$ accelerates with the increase in the magnitude of $\bar{F_{RS}}$ until the force $\bar{F_{RS}}$ reaches a larger magnitude.

## 4. Results

This section details the testing of the proposed approach under various scenarios, utilizing MATLAB R2021a for all simulation studies. The swarm robots are initially dispersed randomly within the environment. Parameters for these simulations are set as follows: A=15, B=1, D=1, μ=1, E=40, σ=−0.8, $r_{0}=\sqrt{2}$, $R_{d}=5$, and $V_{max}=1.4$. The environment is represented by a neural network which has 70×70 neurons. In the simulation, the position of the *pilot* robot and *foraging* robot are denoted by yellow and red points, respectively. The obstacle position is denoted by a black square and the trajectory of the *foraging* robot is shown as the blue line.

### 4.1. Single Target without Sub-Group Behavior

**Figure 4:**
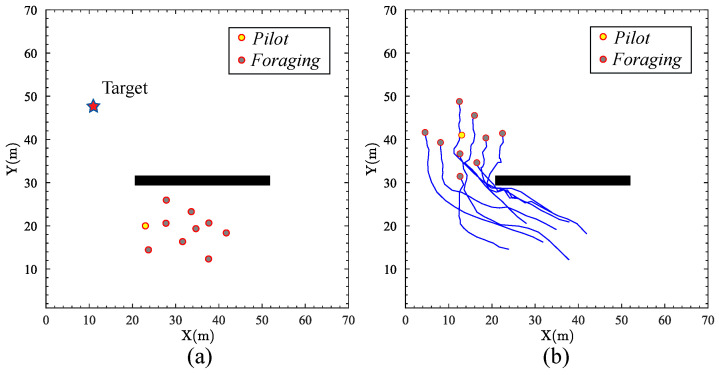
The scenario of a single target without sub-groups: (**a**) The initial positions of the robots and the target. (**b**) The final foraging trajectories of the swarm robots.

The first simulation aims to test the proposed approach considering the scenario of a single target without sub-groups. Figure 4a shows the initial positions of the robots with respect to the target, indicating a singular *pilot* robot and multiple *foraging* robots. The position of the target is (11,49). The positions of the robots are (29,25), (26,16), (37,17), (30,19), (36,24), (34,15), (44,16), (40,10), and (40,23), respectively. Furthermore, an obstacle is located within a specified region, defined by the horizontal coordinates spanning from 20 to 50 and the vertical coordinates ranging from 30 to 31. Figure 4b shows the paths taken by the swarm robots from their initial positions to forage the target. The results demonstrate a collective movement pattern similar to a biological fish school. The *pilot* robot can effectively navigate toward the target while the *foraging* robots follow, maintaining the desired distance. Note that the swarm intelligently maneuvers around an obstacle without compromising its formation integrity or target trajectory. The foraging behavior underscores a high degree of spatial awareness and the capacity to plan dynamic routes within the robotic swarm. The adaptability of the formation in response to environmental obstacles suggests a robust algorithmic foundation that balances individual robot autonomy with collective behavior.

### 4.2. Multiple Targets with Sub-Group Behavior

**Figure 5:**
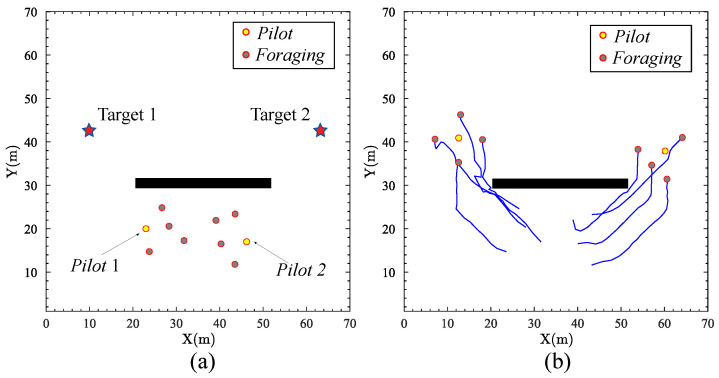
The scenario of multiple targets with sub-group behavior: (**a**) Two distinct groups can be discerned in the swarm robots. (**b**) The final trajectory of the swarm robots.

In the next simulation, the proposed approach is tested considering the scenario of multiple targets with sub-group behavior. The positions of robots obstacle are similar to the previous simulation. However, there are two targets in the environment. The positions of the targets are (11,41) and (62,41). Figure 5 illustrates the strategic deployment of a robotic swarm in an environment with two targets based on the application of the SOM algorithm. Figure 5a shows that the swarm robots are discerned into two distinct groups, each led by a designated *pilot* robot, with the remaining robots designated as *foraging* robots. Figure 5b shows the final trajectory of the swarm robots. The swarm robots are divided into two sub-groups, each foraging a separate target. The trajectories show successful navigation around the obstacle without disrupting the integrity of the sub-group robots or its objective-oriented movement. The observed behavior in the simulation results is similar to fish foraging strategies, where the school of fish disperses into smaller shoals for effective resource exploitation. The robotic swarm exhibits an adaptive division on moving to multiple targets based on the SOM algorithm. The capacity of the robotic swarm to dynamically reconfigure in response to the multiplicity of targets without the loss of efficiency is a testament to the robustness of the proposed approach.

### 4.3. New Target in Sudden-Change Environments

**Figure 6:**
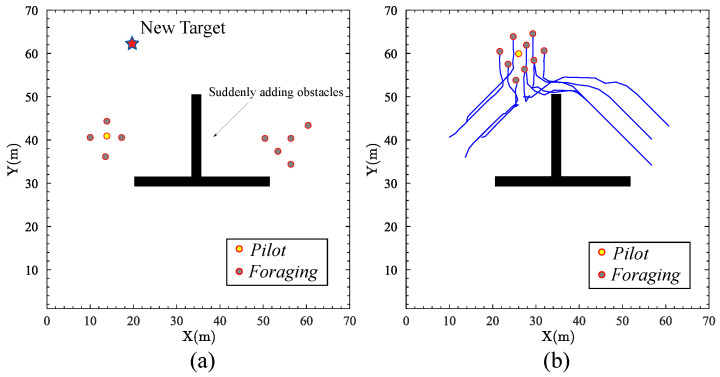
The scenario of a new target in sudden-change environments: (**a**) A new target and obstacle suddenly exist in the environment. (**b**) The trajectories as the swarm robots exhibit a reconfiguration into a consolidated group and are oriented towards the new target.

In this simulation, the proposed approach is tested considering the scenario of a new target in sudden-change environments. Figure 6a shows the swarm robots in the immediate aftermath of the successful foraging with two targets. At that time, a new target and obstacle suddenly exist in the environment. The position of the new target is (21,61). In addition, a sudden-change obstacle is located within a specified region, defined by the horizontal coordinates spanning from 32 to 33 and the vertical coordinates ranging from 31 to 50. Figure 6b shows the trajectories as the swarm robots exhibit a reconfiguration into a consolidated group and are oriented towards the new target. The proposed approach enables swarm robots to reconvene into a singular group after successful sub-group foraging. The trajectory of the swarm robots was unobstructed by the sudden addition of obstacles. The simulation results show a parallel to fish foraging behavior, where schools exhibit a dynamic dispersion for resource acquisition and aggregation for collective objectives. The proposed approach shows a similar ability to disband into sub-groups for task-specific operations, and subsequently amalgamate in the face of new objectives, which underscores the flexibility and dynamic resource allocation of the proposed approach.

### 4.4. Comparison Studies

To evaluate the performance of the proposed approach, a total of 20 test cases are conducted in each evaluation. In these tests, the positions of robots, targets, and obstacles are randomly distributed in the environment. The proposed approach is compared with a novel FIRA method [25]. This method is also a fish-inspired method and achieves similar foraging behavior as the proposed approach, making it a valuable point of comparison. The foraging behavior of the FIRA can be denoted by [25]

(20) $$F_{f}=C_{f}\sum_{j=0}^{m}\left(\left(f_{P_{i}}+P_{t}f_{v_{i}}\right)\frac{D_{f}}{D_{t}}\right)$$

where *m* is the number of food sources; $C_{f}$ is the coefficient of foraging weighting; $f_{P_{i}}$ is the target position; $P_{t}$ is the foraging time; $f_{v_{i}}$ is the velocity of the target; $D_{f}$ is the coefficient of the target attraction; and $D_{t}$ is the distance between the robot and the target. The general obstacle avoidance can be denoted by

(21) $$F_{o}=C_{o}\sum_{j=0}^{p}\left(\left(-f_{P_{o}}+P_{t}f_{v_{o}}\right)\frac{D_{o}}{D_{obs}}\right)$$

where *p* is the number of obstacles; $C_{o}$ is the coefficient of general avoidance weighting; $f_{P_{o}}$ is the obstacle position; $f_{v_{o}}$ is the obstacle velocity; $D_{o}$ is the coefficient of the repulsion forces from obstacle; and $D_{obs}$ is the distance between the robot and the obstacles. In addition to foraging behavior, FIRA incorporates other behaviors, with the final action position being the cumulative result of various behavior vectors. Concurrently, these behaviors are assigned different priorities; for instance, obstacle avoidance is prioritized over foraging behavior. The comparison results are summarized in Table 1.

The proposed method forages multiple targets with shorter distances and less time expended compared to FIRA, as shown in Table 1. This is because FIRA lacks sub-group behavior, as shown in Equation (20). This implies that for multi-target foraging, swarm robots need to traverse through each target point, significantly increasing both the distance and the time required for foraging. In contrast, the proposed method intelligently divides the robot swarm into distinct sub-groups based on the SOM algorithm. These sub-groups concurrently forage on different targets to markedly enhance efficiency. In addition, obstacle avoidance in FIRA is based on a fully connected potential field method. The selection of influence parameters between robots and obstacles presents a significant challenge. Although the impact of obstacles increases as the distance decreases, the repulsive force exerted by obstacles still affects the movement efficiency of the robots even when they are not in close proximity. In contrast, the proposed method features only local connections between neurons, resulting in the global influence of obstacles being confined to a very small area.

## 5. Discussion

The proposed approach aimed to enable robotic systems with the foraging ability of fish. Therefore, most parameters are decided by the task requirements of the foraging, such as the robot velocity and the parameters of the SOM algorithm. The parameters of the shunting equation have been discussed in previous work [31]. In this discussion, the most important parameters *A* and μ are discussed, and some simulations are carried out to demonstrate the effect of these parameters.

**Figure 7:**
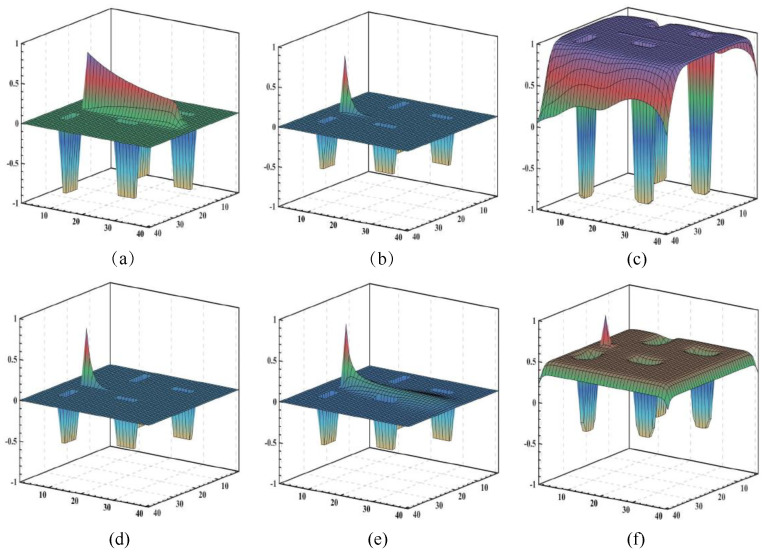
The neural activity when choosing different values. (**a**) A=10. (**b**) A=50. (**c**) Neural network is saturated when A=0.2. (**d**) μ=0.1. (**e**) μ=5. (**f**) Neural network is saturated when μ=10.

The parameter *A* plays a significant role in the transient response of neural activity. In order to investigate the parameter *A*, several simulation experiments are tested with the same parameter settings, except that *A* has a different value. Figure 7 shows three typical results from the simulations varying the parameter *A*. The neural network architecture is 40×40 neurons. The *x*- and *y*-axes are the position of the neuron in the Cartesian workspace. The *z*-axis is the value of the neural activity. The past travel trajectory of the excitatory input has a more lasting influence into the neural network when A=10, as shown in Figure 7a. The small passive damping of the remaining neural activity makes the past influence of the excitatory input disappear slowly. The influence of the past position of the excitatory input on the generation of the trajectory decreases as the value of the parameter *A* increases. This can be observed in the results presented in Figure 7b. The propagation of neural activity within the proposed neural network is observed to be dependent on the current position of the excitatory input. However, it is observed that the neural network may become saturated if a very small value of *A* is selected, as shown in Figure 7c. The saturation causes the robot to find it difficult to distinguish between its current position and its neighboring positions. In summary, it is generally recommended to select a value of *A* within the interval of (0.2,50].

The parameter μ is important in forming the neural activity landscape because the local connection to each neuron is a small region, and the propagation of positive neural activity is able to arrive at the entire neural network. In order to investigate the parameter μ, several simulation experiments with the same parameter settings are tested, except that μ has a different value. The neural activity landscapes in Figure 7b,d are similar, because both systems have the same *A*. However, the propagation of neural activity from the excitatory input is weakened due to choosing a lower μ value. The remaining neural activity has a relatively stronger influence on the neural network. The robot might not choose the optimal neuron for the next movement. The past travel trajectory of the excitatory input in Figure 7e has a much wider influence on the neural network. Compared to the activity landscape in Figure 7a, there is a wide range through which the robot cannot move. Furthermore, when parameter μ>1 it is very easy to saturate the neural activity because the propagated activity is amplified, as shown in Figure 7f. Thus, the value of μ is normally selected to be in the interval μ∈(0,1].

## 6. Conclusions

In this paper, an innovative fish-inspired foraging approach of swarm robots is proposed to forage targets in complex environments. The proposed approach employs a BINN coupled with an SOM algorithm to enable the swarm robots to mimic fish-like patterns such as collision-free movement and dynamic sub-group behavior. The swarm robots are able to reconfigure their movements in response to environmental changes. The foraging process is similar to the versatility and resilience seen in fish foraging. The simulation results indicate enhanced cooperation, efficiency, and adaptability in different scenarios. The proposed approach provided an effective integration of fish-inspired strategies with neurodynamic and swarm intelligence models, which can provide insights for future bio-inspired algorithms and robotic systems. Future work will consider integrating real sensor data to design more intelligent foraging algorithms suitable for increasingly complex and uncertain environments, such as underwater or disaster scenarios. In addition, future work will also apply the proposed approach in real-robot platforms to achieve practical applications, such as pollution detection, search and rescue operations, and biological studies in marine environments.

## Figures and Tables

**Table 1:** Comparison results of the FIRA and proposed method.

Method	Average Travel Distance	Foraging Time
FIRA [25]	58.7 m	41.3 s
Proposed method	27.4 m	23.5 s

## Data Availability

Data are contained within the article.

## References

[1] Ioannou C.C. (2017). Swarm intelligence in fish? The difficulty in demonstrating distributed and self-organised collective intelligence in (some) animal groups. Behav. Process..

[2] Doran C., Bierbach D., Lukas J., Klamser P., Landgraf T., Klenz H., Habedank M., Arias-Rodriguez L., Krause S., Romanczuk P. (2022). Fish waves as emergent collective antipredator behavior. Curr. Biol..

[3] Li J., Yang S.X. (2023). Intelligent escape of robotic systems: A survey of methodologies, applications, and challenges. J. Intell. Robot. Syst..

[4] Li Y., Xu Y., Wu Z., Ma L., Guo M., Li Z., Li Y. (2022). A comprehensive review on fish-inspired robots. Int. J. Adv. Robot. Syst..

[5] Wang T., Yu J., Chen D., Meng Y. (2023). A torque control strategy for a robotic dolphin platform based on angle of attack feedback. Biomimetics.

[6] Chen D., Wu Z., Meng Y., Tan M., Yu J. (2022). Development of a high-speed swimming robot with the capability of fish-like leaping. IEEE/ASME Trans. Mechatronics.

[7] Weber P., Arampatzis G., Novati G., Verma S., Papadimitriou C., Koumoutsakos P. (2020). Optimal flow sensing for schooling swimmers. Biomimetics.

[8] Hannard F., Mirkhalaf M., Ameri A., Barthelat F. (2021). Segmentations in fins enable large morphing amplitudes combined with high flexural stiffness for fish-inspired robotic materials. Sci. Robot..

[9] Zhang F., Pang J., Wu Z., Liu J., Zhong Y. (2023). Effects of different motion parameters on the interaction of fish school subsystems. Biomimetics.

[10] Cioarga R.D., Micea M.V., Cretu V., Groza V. Evaluation of fish shoal inspired movement in collaborative robotic environments. Proceedings of the 2010 IEEE Instrumentation & Measurement Technology Conference Proceedings.

[11] Berlinger F., Wulkop P., Nagpal R. Self-organized evasive fountain maneuvers with a bioinspired underwater robot collective. Proceedings of the IEEE International Conference on Robotics and Automation.

[12] Novák F., Walter V., Petráček P., Báča T., Saska M. (2021). Fast collective evasion in self-localized swarms of unmanned aerial vehicles. Bioinspiration Biomim..

[13] Min H., Wang Z. Design and analysis of group escape behavior for distributed autonomous mobile robots. Proceedings of the IEEE International Conference on Robotics and Automation.

[14] Li J. (2023). Biologically inspired approaches to escape and rescue of multiple robots based on neurodynamic models. Ph.D. Thesis.

[15] Sun B., Li W., Wang Z., Zhu Y., He Q., Guan X., Dai G., Yuan D., Li A., Cui W. (2022). Recent progress in modeling and control of bio-inspired fish robots. J. Mar. Sci. Eng..

[16] Li G., Wong T.W., Shih B., Guo C., Wang L., Liu J., Wang T., Liu X., Yan J., Wu B. (2023). Bioinspired soft robots for deep-sea exploration. Nat. Commun..

[17] Luo J., Qian Z., Gui L., Geng X. (2022). Design and implementation of hybrid autonomous robotic fish platform for underwater emergency search and rescue. Proceedings of the ISCTT 2022: 7th International Conference on Information Science, Computer Technology and Transportation.

[18] Shao J., Wang L., Yu J. (2008). Development of an artificial fish-like robot and its application in cooperative transportation. Control Eng. Pract..

[19] Song Y., Fang X., Liu B., Li C., Li Y., Yang S.X. (2020). A novel foraging algorithm for swarm robotics based on virtual pheromones and neural network. Appl. Soft Comput..

[20] Pang B., Zhang C., Song Y., Wang H. Self-organized task allocation in swarm robotics foraging based on dynamical response threshold approach. Proceedings of the 2017 18th International Conference on Advanced Robotics (ICAR).

[21] Lee W., Vaughan N., Kim D. (2020). Task allocation into a foraging task with a series of subtasks in swarm robotic system. IEEE Access.

[22] Neshat M., Sepidnam G., Sargolzaei M., Toosi A.N. (2014). Artificial fish swarm algorithm: A survey of the state-of-the-art, hybridization, combinatorial and indicative applications. Artif. Intell. Rev..

[23] Li X. (2003). A new intelligent optimization-artificial fish swarm algorithm. Doctor Thesis.

[24] Pourpanah F., Wang R., Lim C.P., Wang X.Z., Yazdani D. (2023). A review of artificial fish swarm algorithms: Recent advances and applications. Artif. Intell. Rev..

[25] Connor J., Joordens M., Champion B. (2023). Fish-inspired robotic algorithm: Mimicking behaviour and communication of schooling fish. Bioinspiration Biomim..

[26] Berlinger F., Gauci M., Nagpal R. (2021). Implicit coordination for 3D underwater collective behaviors in a fish-inspired robot swarm. Sci. Robot..

[27] Zhu D., Cao X., Sun B., Luo C. (2017). Biologically inspired self-organizing map applied to task assignment and path planning of an AUV system. IEEE Trans. Cogn. Dev. Syst..

[28] Grossberg S. (1988). Nonlinear neural networks: Principles, mechanisms, and architectures. Neural Netw..

[29] Hodgkin A.L., Huxley A.F. (1952). A quantitative description of membrane current and its application to conduction and excitation in nerve. J. Physiol..

[30] Li J., Yang S.X., Xu Z. A survey on robot path planning using bio-inspired algorithms. Proceedings of the IEEE International Conference on Robotics and Biomimetics.

[31] Li J., Xu Z., Zhu D., Dong K., Yan T., Zeng Z., Yang S.X. (2021). Bio-inspired intelligence with applications to robotics: A survey. Intell. Robot..

[32] Zhao H., Liu H., Leung Y.W., Chu X. (2018). Self-adaptive collective motion of swarm robots. IEEE Trans. Autom. Sci. Eng..

